# External Fixators as a Tool for Damage Control Orthopedics in Severely Injured or Polytrauma Patients

**DOI:** 10.7759/cureus.69255

**Published:** 2024-09-12

**Authors:** Ismini Kountouri, Panagiotis Christidis, Georgios Christidis, Georgios Biniaris, Nikolaos Gougoulias

**Affiliations:** 1 Department of General Surgery, General Hospital of Katerini, Katerini, GRC; 2 Department of Orthopedics and Traumatology, General Hospital of Katerini, Katerini, GRC

**Keywords:** damage control orthopaedics, early total care, external fixation, polytrauma, retrospective cohort study

## Abstract

The purpose of this study is to explore the use of damage control techniques in the emergency surgical management of polytrauma patients - those with traumatic injuries affecting at least two anatomical regions - at a District General Hospital in Greece. We conducted a retrospective review of medical records from patients who visited the orthopedic emergency department between 2021 and 2024. From approximately 10,000 injured patients treated annually in our emergency department, we selected a sample of 29 polytrauma patients who required surgical intervention. We utilized the Injury Severity Score (ISS) to evaluate these patients. For 16 patients, the initial surgical intervention was also the definitive treatment, utilizing intramedullary nailing or internal osteosynthesis techniques. In the remaining 13 patients, damage control techniques, including external osteosynthesis (ExFix), were employed. The ISS was the primary criterion for deciding between definitive management and damage control procedures. Data on the 13 patients managed with damage control techniques were further analyzed and are presented in this study. External osteosynthesis was used to stabilize fractures and control bleeding, particularly in patients with multiple orthopedic injuries such as femoral or diaphyseal tibial fractures. This approach facilitated resuscitation and recovery. Our findings suggest that stabilizing long bone fractures with external fixation in patients with an ISS greater than 9 is both safe and likely contributes to overall recovery. This study demonstrates that a damage control approach for polytrauma patients with significant orthopedic trauma is effective for fracture stabilization and bleeding control. Additionally, in three cases, this approach also served as the definitive treatment.

## Introduction

Polytrauma patients are defined as those with injuries affecting at least two anatomical regions of the body [1]. According to AO scholars, a polytrauma patient with an Injury Severity Score (ISS) greater than 17 is considered severely injured and at risk of systemic organ failure and death [1]. The Berlin definition of polytrauma characterizes it as a patient with an Abbreviated Injury Scale (AIS) score of 3 or higher in two or more different body regions, along with one or more additional variables from the five physiological parameters [2].

Polytrauma patients with multiple orthopedic injuries benefit significantly from the early fixation of major long bone fractures, although the optimal timing and type of fixation remain subjects of debate [3]. The term “damage control” refers to an initial, limited surgical intervention in severely injured patients designed to mitigate the impact of surgery, avoid the second hit phenomenon, and ensure immediate survival [4]. The second hit phenomenon suggests that further trauma from unstable fractures and definitive fixation surgery can lead to systemic inflammatory response syndrome, multiple organ dysfunction syndrome, or multiple organ failure (MOF) [1].

Damage control orthopedics (DCO) techniques are employed in polytrauma patients with significant musculoskeletal trauma to enable rapid bone stabilization (e.g., external fixation), reduce the risk of a second hit phenomenon caused by more invasive surgeries (e.g., intramedullary reaming or internal fixation, which can lead to bleeding and soft tissue damage) [3], and address the critical threats of metabolic acidosis, hypothermia, and coagulation disorders resulting from hemorrhagic shock [1]. Conversely, the early total care (ETC) approach involves the primary definitive treatment, such as intramedullary nailing or plate fixation, which can potentially trigger the second hit phenomenon [1].

This study aims to present the application of DCO management for polytrauma patients with predominantly orthopedic injuries at a District General Hospital in Katerini, Greece.

## Materials and methods

We retrospectively reviewed the medical records of 29 polytrauma patients treated at the Orthopedics Department of the General Hospital of Katerini from March 2021 to April 2024. Among these patients, 21 were male and eight were female, with an average age of 47.0 ± 21.2 years (range: 17-90). The causes of their injuries varied: 20 patients were involved in motor vehicle accidents, seven suffered falls, one was crushed under a heavy object, and one was a gunshot victim.

To assess the severity of trauma, we used the ISS. According to AO scholars, severe polytrauma is defined as having an ISS greater than 17, indicating a high risk of systemic organ failure and death [1]. The ISS is an anatomical scoring system for patients with multiple injuries, ranging from 0 to 75, and is based on the AIS [5,6]. The AIS score uses an ordinal scale from 1 to 6 to categorize injuries into six body regions: neck, face, thorax, abdomen and pelvic cavity, extremities and pelvis, and body surface. Each region is assigned an AIS score (Table 1) [1].

**Table 1:** AIS In the AIS, the body is divided into six anatomical regions, and each region is assigned an AIS score [1,5,6]. AIS: Abbreviated Injury Scale

Score	AIS
1	Minor
2	Moderate
3	Serious
4	Severe
5	Critical
6	Unsurvivable

The ISS is calculated using the AIS values of the three most severely injured anatomical regions [6]. If any injury is assigned an AIS score of 6, the ISS is automatically set to 75, indicating an unsurvivable injury [6-8]. The ISS score allows for the categorization of trauma into different severity levels: mild (ISS <9), moderate (ISS 9-15), severe (ISS 16-24), or profound (ISS ≥25) [5].

## Results

Using the ISS, our patients were categorized as detailed in Table 2. Specifically, eight patients were classified with mild trauma, eight with moderate trauma, nine with severe trauma, and four with profound trauma. For the management of these patients, 16 received primary intramedullary nailing and/or internal osteosynthesis techniques, representing definitive treatment. In contrast, 13 patients were managed with damage control techniques, including external osteosynthesis (ExFix). The patients treated with definitive methods were classified as the ETC group, while those managed with damage control techniques were categorized as the damage control (DC) group.

**Table 2:** Patient categorization using the ISS ETC: early total care; DCO: damage control orthopedics; ISS: Injury Severity Score; MVA: motor vehicle accident

		Mild trauma	Moderate trauma	Severe trauma	Profound trauma	
ISS, mean ± SD		5.1 ± 1.6	10.5 ± 1.9	19.14 ± 1.8	40.7 ± 10.3	
Gender, n	Female	3/8	2/8	2/9	2/4	
	Male	5/8	6/8	7/9	2/4	
Age, mean ± SD (years)		34.1 ± 21.2	48.7 ± 16.8	58.0 ± 15.4	53 ± 25.2	
Cause, n	MVA	6/8	4/8	7/9	3/4	
						Fall	2/8	3/8	2/9	0/4	
						Gunshot	0/8	0/8	0/9	¼	
	Crushed	0/8	1/8	0/9	0/4	
	Intubation in the ED, n		0	0	1/9	3/4	
	ICU		0	1/8	2/9	3/4	
Days of hospitalization, mean ± SD		10.5 ± 2.8	3.8 ± 6.1	17.5 ± 11.4	13 ± 2.7	
						Transferred to tertiary/university hospitals		0/8	0/8	2/9	3/4	
Type of management	ETC	8/8	6/8	2/9	0/4	
						DCO	0/8	2/8	7/9	4/4	

Patients in the ETC group had an average ISS of 8.6 ± 4.5, while those in the DC group had a significantly higher average ISS of 24.5 ± 12.6. A detailed analysis was conducted on the patients who underwent damage control orthopedic surgery. External fixation was employed for eight tibial fractures, three femoral fractures, one elbow bridging for a supracondylar humerus fracture, and one unstable pelvic injury. Descriptive statistics for the DC group are presented in Table 3.

**Table 3:** DCO patients’ descriptive statistics ACLR: anterior cruciate ligament rupture; AVN: avascular necrosis; B: bilateral; C: cervical; DCO: damage control orthopedics; ETC: early total care; EXFIX: external fixation; FX: fracture; GA: Gustillo-Anderson; GSW: gunshot wound; I&D: irrigation and debridement; IMN: intramedullary nail; ISS: injury severity score; MC: metacarpal; MT: metatarsal; MVA: motor vehicle accident; OA: osteoarthritis; ORIF: open reduction and internal fixation; PF: percutaneous fixation; R: right; T: thoracic; UTI: urinary tract infection; VAC: vacuum assisted closure

Gender	Age	Injuries	ISS	Cause	Initial care	Final care	Duration between initial and final care (days)	ICU stay (days)	Hospital stay (days)	Complications
F	17	Closed pelvic fx (“open book”), T2-T4 fx, L2-L3 fx, degloving injury of the R foot	25	MVA	ExFix for pelvis fx (one hour after arrival at the ER)	No further treatment regarding the pelvis; skin coverage by plastics for degloving injury elsewhere; no bracing method was required	-	-	11	-
M	50	R open (GA-II) distal tibial fx, L open ankle dislocation	18	MVA	Uniplanar ExFix for B tibial fx (30 minutes after arrival at the ER)	Transferred to tertiary hospital for definitive treatment after initial stabilization	-	-	6	-
M	55	R closed distal femoral fx w intra-articular involvement, L closed diaphyseal femoral fx	9	MVA	ORIF for R femur fx and uniplanar ExFix for L femur fx (three hours after arrival at the ER)	IMN for L femur	19	9 days	19	Hardware breakage (screws) in R femur a year later (removal)
M	42	R closed distal femoral fx, L open (GA-IIIa) diaphyseal tibial fx	18	MVA	Uniplanar ExFix for tibial fx (three hours after arrival at the ER)	ORIF of R femur and IMN of L tibial	25 days for tibial fx; four days for femoral fx	-	15	-
M	44	R open (GA-II) distal tibial fx w intra-articular involvement, L closed calcaneal fx	18	MVA	Multiplanar ExFix for tibial fx and PF for calcaneum (one hour after arrival at the ER)	No further treatment	-	-	18	-
M	69	L open (GA-II) distal tibial fx w intra-articular involvement, L closed diaphyseal clavicular fx, L 8-9th rib fx	22	Fall	Uniplanar ExFix for tibial fx, sling (30 minutes after arrival at the ER)	Ankle fusion w plate	8	-	16	Deep wound infection after the definitive treatment; UTI
M	47	R open (GA-I) distal humeral fx, open abdominal and thoracic injuries ( mesenteric ischemia and multiple lung contusions	50	GSW	Uniplanar ExFix for humeral fx and laparotomy (three hours after arrival at the ER, immediately after the laparotomy)	Transferred to a tertiary hospital after initial stabilization for a cardiothoracic team in order to receive definitive treatment for the thoracic injuries	-	Two days	2	Septic shock in the ICU recovered and was transferred after initial stabilization
M	56	L open (GA-II) distal femoral fx w intra-articular involvement and communication	13	Fall	Uniplanar ExFix for femoral fx (one hour after arrival at the ER)	ORIF	21	-	28	Deep wound infection (I&D) after the definitive treatment; hardware breakage months after the ORIF
M	53	L closed distal femoral and L proximal tibial fx w intra-articular involvement (“floating knee”), L closed ankle fx, L scapular fx, C7-T2 fx, thoracic cage contusion	18	MVA	Bridging ExFix, cervical brace (one hour after arrival at the ER)	Transferred to tertiary hospital after initial stabilization in the ICU	-	10 days	10	-
F	87	R closed proximal femoral fx, R open (GA-IIIa) diaphyseal tibial fx, L scapular fx, T2-T3 fx, T11fx, retropharyngeal hematoma, pulmonary and brain contusion	38	MVA	Long G-nail and uniplanar ExFix for tibial fx (12 hours after arrival at the ER)	Transferred to tertiary hospital after initial stabilization in the ICU	-	10 days	10	-
M	42	B open (GA-II) diaphyseal tibial fx, R closed diaphyseal humeral fx	18	MVA	ExFix for B tibial fx, cast for humerus (three hours after arrival at the ER)	No further treatment	-	15 days	44	-
M	61	L open (GA-II) distal femoral fx, R closed distal femoral fx w intra-articular involvement, L closed shoulder dislocation, closed pelvic fx, T2 fx, multiple L rib fx, mesenteric hemorrhage	50	MV	Bridging ExFix for B femoral fx, exploratory laparotomy (three hours after arrival at the ER, immediately after the laparotomy)	IMN for L and R femur	60	40 days	37	Septic shock in the ICU two days after the initial trauma
M	72	R open (GA-I) distal tibial fx, R medial malleolar fx, R zygomatic fx	22	MVA	multiplanar ExFix for tibial fx and PF for malleolar fx (one hour after arrival at the ER)	IMN	28	-	14	Loss of reduction (10 days after initial DCO)

All patients survived their injuries, were discharged from the ICU, and eventually returned home. Some required extended rehabilitation at specialized centers. Notably, one patient died several months after the injury due to a cause unrelated to the initial trauma - a heart attack that occurred a year later while dancing at a wedding.

Among the 13 patients who underwent DCO, five received definitive surgical treatment with internal osteosynthesis or intramedullary nailing at a later stage (between seven and 60 days post-injury). Four of these patients were transferred to tertiary hospitals for further treatment due to their general condition, while three patients for whom external fixation was initially applied continued with this method as their definitive treatment.

In total, six of the 29 patients were admitted to the ICU, and all of these were from the DC group. The ICU admissions included one patient with moderate trauma (ISS = 9), two with severe trauma (ISS = 18 and 18), and three with profound trauma (ISS = 50, 38, and 50). No patients treated with ETC methods were admitted to the ICU.

Our results indicate that patients with higher ISS values were more likely to be treated with DCO techniques, whereas those with lower ISS values received ETC methods. Interestingly, all patients with ISS <9, indicating mild trauma, were treated with ETC techniques, while all patients with ISS ≥25, indicating profound trauma, were managed with DCO techniques.

Typical cases of damage control surgery within our cohort of polytrauma patients are presented below.

Case 1

A 53-year-old patient was brought by ambulance to the emergency department of the General Hospital of Katerini following a car accident. Initial evaluation in the emergency room revealed no airway obstruction; however, the patient exhibited decreased breath sounds on the left side of the lung and a blood oxygen level drop to 85%. His blood pressure and heart rate were within normal limits, and his Glasgow Coma Scale (GCS) score was 15 out of 15. The patient complained of severe dyspnea, intense pain in the left thigh and knee, and discomfort in the left shoulder. Suspected injuries included femoral and tibial fractures, as well as a tension pneumothorax.

An initial needle decompression was performed, followed by the insertion of a thoracic tube approximately two minutes after arrival in the shock room. This intervention quickly stabilized his SpO2 levels and controlled the dyspnea. A central IV line was placed, and intravenous fluids and analgesics were administered. With the patient hemodynamically stable, a full-body CT scan and X-rays of the extremities were conducted, revealing a left supracondylar femur fracture, a left tibial condyle fracture, a left ankle fracture, multiple spinal (C7-T2) and rib fractures, a left pneumothorax with a thoracic tube in place, and a left scapula fracture. No intra-abdominal or intracranial bleeding was detected.

**Figure 1:**
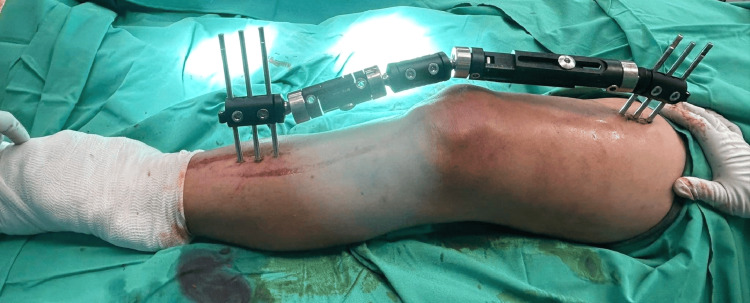
Bridging ExFix between the patient’s left femur and tibia for stabilization of the left tibial condyle fracture

The patient was then intubated and promptly taken to the operating room for knee-bridging external fixation to stabilize the left tibial condyle fracture (Figure 1).

The patient remained in the ICU for the weekend. Due to ongoing respiratory distress and the need for specialized cardiothoracic and pulmonary evaluations, which were not available at our hospital, he was subsequently transferred intubated to a tertiary hospital for further treatment.

Case 2

An 87-year-old woman was brought to the emergency department by ambulance after being struck by a car. Upon initial evaluation, she had no airway obstruction and normal respiratory sounds. There was no circulatory disruption, but her GCS score was 13/15. She presented with an open wound and deformity in the right tibia and reported significant pain in her right lower extremity, left shoulder, and back. Although she was hemodynamically stable at first, she was intubated in the emergency room. A central line was inserted, and IV fluids and antibiotics were administered.

Before radiological imaging, the open tibial fracture was irrigated with 3 liters of normal saline and temporarily stabilized with a splint. Subsequently, a full-body CT scan and X-rays of her extremities were performed. The imaging revealed right subtrochanteric femoral and right tibial fractures, a left scapula fracture, a postero-trancheal hematoma, and multiple pulmonary and hemorrhagic brain contusions.

**Figure 2:**
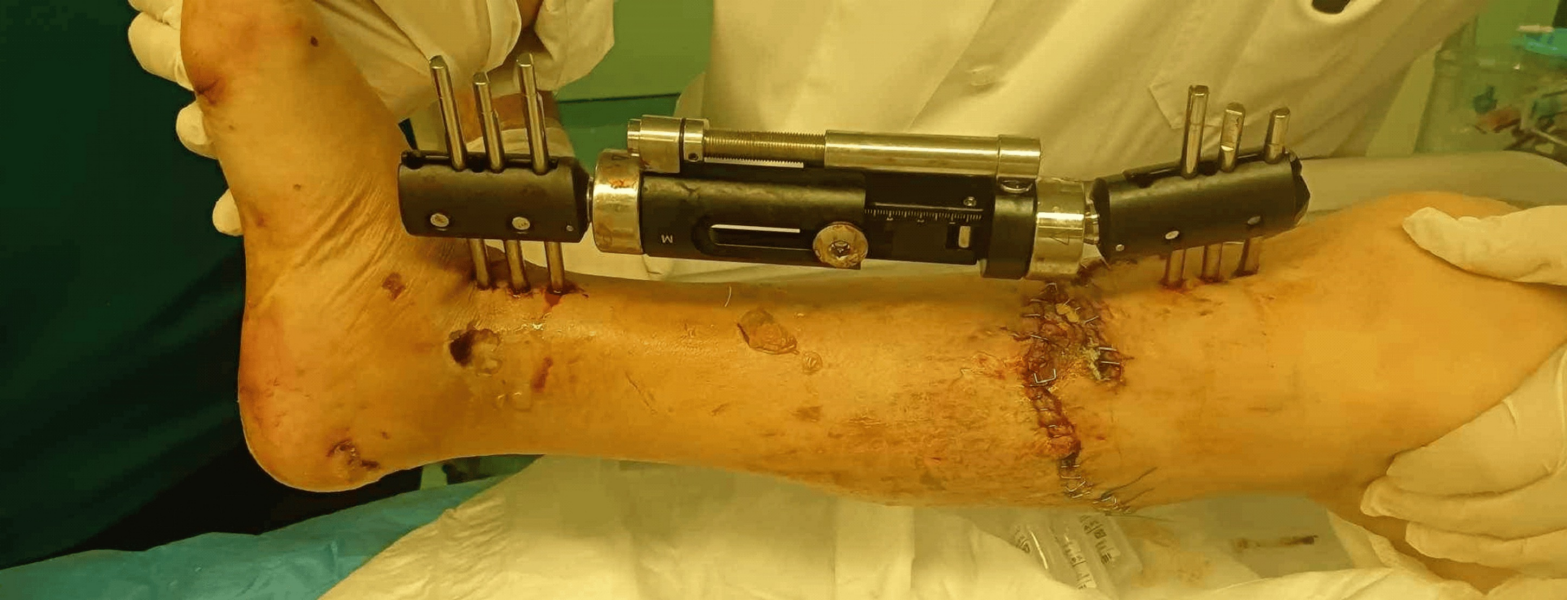
ExFix on the patient’s right tibia

The following day, the patient underwent surgery for external fixation of the right tibia and suturing of the open wound (Figure 2). Additionally, an intramedullary nail (G-nail) was inserted to stabilize the right femoral subtrochanteric fracture. The decision to perform early internal stabilization for femoral fractures within the first 24 hours is generally supported, even in patients with chest trauma [3]. However, the potential for the second hit phenomenon, where early fixation might exacerbate trauma effects, remains controversial. In stable polytrauma patients, early reamed femoral nailing is considered acceptable. For unstable patients, decision-making is complex and influenced by factors such as initial trauma severity, resuscitation response, and individual patient characteristics [3]. In this case, early stabilization was deemed safe and beneficial, allowing for recovery and stability in the ICU. The patient remained in the ICU for a month, underwent a tracheostomy, and was eventually transferred to a rehabilitation center for further recovery.

This case exemplifies how an initial damage control approach ultimately became the definitive treatment for the patient.

Case 3

A 17-year-old girl was brought to the emergency department following a motorcycle accident, where she was a passenger. On initial evaluation, she had no airway obstruction, her vital signs were within normal limits, and she experienced no breathing or circulation difficulties. Her GCS score was 15 out of 15, although she reported pain in her right pelvic region and had a degloving injury on the dorsum of her right foot.

The degloving injury was treated with extensive irrigation and debridement to remove debris, followed by reattachment of the avulsed skin and subcutaneous tissue to achieve immediate closure of the wound. Despite the injury, the patient was hemodynamically stable. X-rays revealed pubic symphysis diastasis and a right acetabular fracture, confirming an unstable pelvis. A full-body CT scan showed no intra-abdominal or retroperitoneal bleeding but did reveal fractures at T3-T4 and L2-L3.

**Figure 3:**
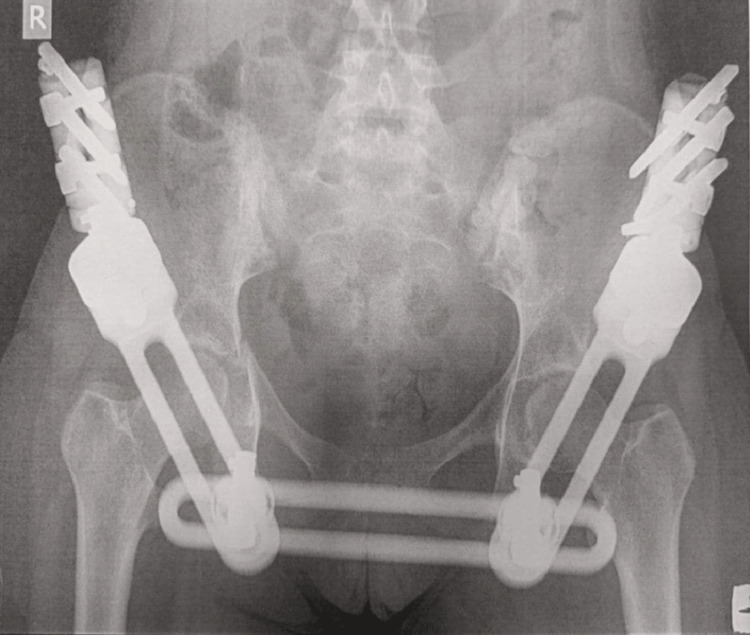
ExFix application on the pelvis

In the operating room, an external fixator was applied to stabilize and reduce the pelvis (Figure 3). The patient did not require ICU care and stayed in the orthopedic ward for two days before being transferred to a tertiary hospital for specialized management of her foot injury by plastic surgeons. No additional treatment was needed for the vertebral fractures or acetabular fractures.

The external fixator was removed six weeks after the injury, and the patient showed no need for further treatment related to her fractures. Three years later, she remains in excellent condition. This case illustrates how the initial DCO approach effectively served as the definitive treatment, leading to a successful recovery for this young patient.

## Discussion

In our cohort of polytrauma patients primarily experiencing musculoskeletal injuries, treated at a secondary care hospital, either DCO or ETC strategies were employed based on the severity of the injuries. All patients recovered from their injuries and were discharged home with minimal complications.

According to the two hits theory, a severe injury constitutes the first hit, while the second hit involves complications such as respiratory distress with hypoxia, repeated cardiovascular instability, metabolic acidosis, ischemia/reperfusion injuries, dead tissue, contaminated catheters or tubes, and infections [8]. Surgical interventions can further contribute to the second hit through severe tissue damage, hypothermia, blood loss, inadequate or delayed surgical or intensive care, and massive transfusions [8].

In polytrauma patients with multiple orthopedic injuries, the DCO approach focuses on rapid bone stabilization to minimize the risk of the second hit phenomenon, which can occur due to major surgery in an already traumatized patient [3,9]. Our approach aims to stabilize fractures to control bleeding, thus preventing hypovolemic shock and the lethal triad [9]. Skeletal fractures, such as those of the femur, pelvis, or lower limbs, are significant sources of blood loss and must be managed carefully in polytrauma cases requiring resuscitation [9].

At our district (secondary care) hospital in Katerini, Greece, we frequently manage polytrauma patients with severe or profound injuries. Our facility, the primary hospital in a tourist area near a highway, deals with numerous motor vehicle accidents, particularly during the summer months.

Our hospital includes a surgical and an orthopedics department (30 beds each) and an ICU with 12 beds. Without neurosurgeons, pediatric surgeons, or cardiovascular surgeons, we often need to transfer patients to central hospitals an hour away. Thus, prompt decision-making is crucial for managing injuries that require emergency intervention. Typically, abdominal-thoracic trauma is addressed first, followed by the application of DCO methods.

Originally, the DCO concept focused on long bone fractures, particularly the femur. However, it has since expanded to include pelvic fractures, spine fractures, and upper limb injuries [9]. Femoral fractures can be significant sources of blood loss [9]. To manage this, external fixation can be employed to immobilize the fracture and control bleeding in polytrauma patients [9]. However, for severely injured patients, alternative techniques such as early intramedullary nailing, non-compressive garments, or skeletal traction might be preferred by some [9,10]. Regardless of the method, femoral fractures must be treated as potentially life-threatening injuries, akin to intra-abdominal bleeding [9].

In cases of unstable pelvic fractures, the DCO approach is widely accepted as the preferred treatment [11] to prevent hemodynamic instability and maintain blood volume and tissue oxygenation. Immediate external fixation and pelvic packing are recommended to control hemorrhage, with angiographic embolization reserved for more stable patients [11]. Definitive pelvic ring reconstruction is considered only after hemorrhage is controlled and the patient is adequately resuscitated [12].

Several studies align with our findings. Wang et al. (2008) retrospectively examined 53 polytrauma patients primarily with orthopedic injuries, where DCO was used. They found that DCO, utilizing external fixation, was safe and resulted in all patients surviving. Among these patients, 38 returned to their previous employment, 11 managed daily life independently, and four required further treatment [1].

Tuttle et al. (2009) compared polytrauma patients with femoral shaft fractures treated with ETC and DCO. They assessed outcomes such as mortality, pulmonary complications, transfusion requirements, and MOF. No significant differences were found between the groups in terms of ARDS, lung scores, MOF, ICU length of stay, or hospital length of stay [10].

Caba-Doussoux et al. (2012) reviewed 41 polytrauma adults with femoral fractures who received external fixation within 12 hours of trauma. They observed a 30% rate of ARDS and six cases of MOF, with five patients dying. They concluded that an aggressive and early DCO approach resulted in a low mortality rate [3].

von Lübken et al. (2023) compared DCO and ETC using data from the German Trauma Society (TraumaRegister DGU®). They found that DCO was more likely in patients with penetrating trauma, packed red blood cell transfusions, unstable pelvic fractures, and lower extremity injuries. However, they did not detect significant benefits of DCO regarding reduced complications or mortality [4].

These studies support the safety and effectiveness of the DCO approach in polytrauma patients, particularly those with a higher ISS.

## Conclusions

Our study demonstrates that the damage control approach, utilizing external fixation for long bone or pelvic fractures in severely injured polytrauma patients (with ISS >16), is both safe and effective. In our cohort, none of the patients succumbed to their injuries, and only a few required transfer to tertiary centers. Rapid stabilization of long bone fractures, typically within six hours of injury, likely helped prevent secondary systemic complications and contributed to overall patient recovery.

As there are no clear guidelines or absolute indications for applying DCO techniques and conducting randomized comparative studies in severely injured patients is challenging, we advocate for data collection through multicenter studies. Such research is crucial to better understand the role of damage control orthopedics in both ensuring primary survival and serving as definitive treatment for polytrauma patients.
